# Anterior Cruciate Ligament Reconstruction in Young Athletes: A Comprehensive Review of Lateral Extra-Articular Tenodesis and Anterolateral Ligament Reconstruction Techniques

**DOI:** 10.7759/cureus.70333

**Published:** 2024-09-27

**Authors:** Bhushan Patil, Saksham Goyal, Ankur Salwan, Rahul Singh

**Affiliations:** 1 Orthopaedics, Jawaharlal Nehru Medical College, Datta Meghe Institute of Higher Education and Research, Wardha, IND

**Keywords:** acl injuries, anterolateral ligament reconstruction, knee stability, lateral extra-articular tenodesis, surgical techniques, young athletes

## Abstract

Anterior cruciate ligament (ACL) injuries are prevalent among young athletes and pose significant challenges due to their impact on immediate and long-term knee function. Traditional ACL reconstruction techniques, while effective, may not always meet the unique needs of this population, particularly given their high physical demands and the risk of future complications. This review evaluates two advanced surgical techniques - lateral extra-articular tenodesis (LEAT) and anterolateral ligament (ALL) reconstruction - as potential enhancements to conventional ACL reconstruction. LEAT involves augmenting knee stability by addressing lateral compartment issues, while ALL reconstruction focuses on reconstructing the ALL to improve overall knee function. The review compares these techniques regarding surgical procedures, clinical outcomes, biomechanical effectiveness, and complications. LEAT and ALL reconstruction are examined for their impact on recovery, return-to-sport rates, and long-term knee health, highlighting their advantages and limitations. Findings suggest that both techniques offer promising benefits, with the potential for improved outcomes compared to traditional methods. However, the effectiveness of each technique can vary based on individual factors and the specific demands of different sports. Further research is needed to fully understand the long-term implications and refine these approaches. This review aims to guide clinical decision-making and optimize treatment strategies for young athletes with ACL injuries, enhancing their prospects for a successful return to athletic activity.

## Introduction and background

Anterior cruciate ligament (ACL) injuries are a prevalent and serious concern among young athletes, particularly those participating in high-impact sports such as football, basketball, and soccer [1]. The rising incidence of ACL injuries in this demographic reflects both the increased involvement in competitive sports and the heightened physical demands placed on young athletes. These injuries are not only painful and debilitating but also carry significant long-term consequences [2]. Beyond the immediate effects, such as pain and functional limitations, young athletes often endure extensive rehabilitation periods and face a higher risk of recurrent instability or secondary injuries even after surgical intervention. The potential for early-onset osteoarthritis and other joint-related complications further underscores the importance of effective treatment strategies [3].

The primary objective of ACL reconstruction is to restore knee stability and functionality, allowing athletes to return to their pre-injury activity levels. However, traditional ACL reconstruction techniques may not always meet the specific needs of younger athletes, who often have higher expectations and greater physical demands [4]. Effective treatment is critical not only for addressing the immediate injury but also for ensuring long-term joint health and athletic performance [5]. Advances in surgical techniques, such as lateral extra-articular tenodesis (LEAT) and anterolateral ligament (ALL) reconstruction, offer potential improvements by addressing additional aspects of knee stability that traditional methods may overlook. Evaluating the effectiveness of these techniques is crucial for optimizing treatment protocols and enhancing the overall quality of life for young athletes [5].

This comprehensive review aims to compare and evaluate the effectiveness of LEAT and ALL reconstruction techniques in the context of ACL reconstruction for young athletes. By analyzing the surgical procedures, clinical outcomes, biomechanical considerations, and potential complications associated with these techniques, this review seeks to provide valuable insights into their relative advantages and limitations. The goal is to contribute to a better understanding of how these advanced surgical options impact recovery and long-term knee health, ultimately guiding clinical decision-making and improving treatment strategies for young athletes dealing with ACL injuries.

## Review

Anatomy and biomechanics

The ACL is a crucial stabilizing structure within the knee joint, primarily preventing excessive anterior tibial translation and internal rotation. It comprises two main bundles: the anteromedial (AM) bundle and the posterolateral (PL) bundle. The AM bundle is most active during knee flexion, providing critical stability against anterior tibial displacement. In contrast, the PL bundle is more influential in stabilizing the knee during extension and controlling rotational movements [6]. The ACL is responsible for approximately 85% of the restraining force against anterior tibial translation across various degrees of knee flexion. It underscores its importance for knee stability, particularly during dynamic activities such as cutting, jumping, and rapid deceleration. Additionally, the ACL contributes to the screw-home mechanism of the knee, which enhances stability during terminal extension [7]. The lateral compartment of the knee includes several key structures that contribute to overall knee stability. These include the lateral collateral ligament (LCL), the iliotibial band (ITB), and other ligaments and tendons that support the knee’s lateral aspect [8]. The LCL resists varus stress, while the ITB aids in stabilizing the knee during flexion and extension. A thorough understanding of these lateral structures is essential, particularly in ACL injuries, as damage to the ACL often coincides with injuries to these lateral stabilizers. This interaction can significantly impact the knee’s overall stability and influence surgical decisions related to reconstruction and rehabilitation protocols [8]. The ALL is a recently identified structure that plays a significant role in knee stability, particularly in conjunction with the ACL. Located on the lateral side of the knee, the ALL is thought to assist in controlling rotational stability, especially during pivoting or cutting activities [9]. It enhances knee stability by providing additional restraint against internal rotation and anterolateral translation. Its role becomes particularly crucial in ACL injuries, as it may help compensate for losing stability when the ACL is compromised. Some studies suggest that reconstructing the ALL alongside the ACL can improve postoperative outcomes and reduce the risk of further instability [10].

ACL reconstruction techniques

ACL reconstruction techniques have undergone significant advancements, offering various methods tailored to meet the needs of patients, especially young athletes. This overview will examine traditional ACL reconstruction, LEAT, and ALL reconstruction, focusing on their surgical procedures, indications, contraindications, and effectiveness [11]. Traditional ACL reconstruction generally employs arthroscopic techniques, where a graft replaces the torn ligament. Common grafts include autografts, such as the patellar tendon or hamstring tendons. The primary goal of the procedure is to restore knee stability and function by accurately recreating the ACL’s anatomy [12]. Outcomes of traditional ACL reconstruction can vary, with graft failure rates reported between 10% and 25%. Anatomic reconstruction, which aims to restore the native anatomy of the ACL, has become the gold standard, leading to improved stability and reduced risks of postoperative complications, such as osteoarthritis [13]. LEAT is a supplementary procedure involving the use of the ITB or other tissue to reinforce the lateral aspect of the knee. Often performed alongside traditional ACL reconstruction, LEAT enhances stability, particularly in patients with high instability or those returning to high-demand sports. It is indicated for patients with high-grade ACL injuries, increased risk of graft failure due to high activity levels, or prior knee instability [14]. However, LEAT may not be suitable for patients with isolated ACL tears without additional instability or those with significant comorbidities that could complicate the surgery or recovery. LEAT has been shown to improve knee stability and reduce the risk of graft failure, especially in young athletes. Studies indicate that combining LEAT with ACL reconstruction can result in better functional outcomes and lower rates of postoperative instability. Nevertheless, this procedure does not fully restore the normal anatomy and function of the ACL, which remains a limitation [15]. ALL reconstruction involves the surgical restoration of the ALL, which works with the ACL to stabilize the knee. This procedure, often performed arthroscopically, is typically used as an adjunct to ACL reconstruction, particularly in cases of rotational instability [16]. It is indicated for patients with ACL tears and significant rotational instability or young athletes with high functional demands. ALL reconstruction may not be suitable for patients without significant rotational instability or those with previous knee surgeries, which could complicate the procedure. Research suggests that ALL reconstruction can enhance knee stability and reduce the incidence of graft failure compared to ACL reconstruction alone [17]. Studies report improved functional outcomes and lower re-injury rates with this combined approach. However, long-term outcomes are still being evaluated, and further research is needed to confirm definitive benefits [17-24]. A comparative summary of ACL reconstruction techniques - traditional ACL reconstruction, LEAT, and ALL reconstruction - is provided in Table 1.

**Table 1:** Comparison of ACL reconstruction techniques: traditional ACL reconstruction, LEAT, and ALL reconstruction ACL, anterior cruciate ligament; ALL, anterolateral ligament; LEAT, lateral extra-articular tenodesis

Technique	Surgical procedure	Indications	Contraindications	Outcomes
Traditional ACL reconstruction [25]	Involves harvesting autografts (patellar tendon, hamstring, or quadriceps tendon) or using allografts to replace the torn ACL. The arthroscopic procedure is commonly used.	Indicated for ACL tears, especially in athletes involved in pivoting sports. Suitable for patients seeking to restore knee stability and function.	It is not ideal for patients with extensive damage to surrounding structures or growth plate concerns in young athletes.	High rates of return to sport, good knee stability, but potential for graft failure or injury to other knee structures.
LEAT [26]	An additional procedure combined with ACL reconstruction. Involves reinforcing the lateral structures of the knee to improve rotational stability.	Used for patients with high-grade pivot shifts or hyperlaxity. This is often indicated when extra rotational stability is needed, especially in high-demand athletes.	Contraindicated in patients with growth plate concerns, the procedure involves placing tension on lateral structures.	Provides enhanced rotational stability, potentially reducing re-injury rates in pivoting sports. It may have a longer recovery period compared to ACL reconstruction alone.
ALL reconstruction [27]	Reconstructs the ALL to support rotational stability, often in conjunction with ACL reconstruction.	This is indicated in patients with ACL tears, especially with combined rotational instability. Ideal for patients with ligament laxity and high pivot shift grades.	Growth plate involvement and the risk of excessive tension on the lateral knee structures are concerns, particularly in younger athletes.	Better rotational control and lower re-injury rates. Limited long-term outcome data but promising in enhancing knee stability and reducing graft failure.

Comparison of techniques

The comparison of surgical techniques for ACL reconstruction, with a focus on LEAT and ALL reconstruction, can be assessed across several key dimensions: clinical outcomes, biomechanical considerations, complications and risks, and rehabilitation and recovery [28]. Return-to-sport rates following ACL reconstruction vary significantly depending on the surgical technique used. These rates range from 41% to 100%, with many studies reporting rates exceeding 80% for both LEAT and ALL reconstruction techniques [29]. However, specific outcomes can be influenced by patient demographics and additional knee injuries. Functional outcomes, which evaluate knee stability and performance during athletic activities, are generally favorable for both techniques [30]. Evidence suggests that integrating LEAT or ALL reconstruction may result in improved functional outcomes compared to traditional ACL reconstruction alone, particularly in patients with higher activity levels [31]. Biomechanical studies indicate that both LEAT and ALL reconstruction techniques enhance knee stability. LEAT contributes additional lateral stability, which is particularly effective in preventing rotational instability [32]. In contrast, ALL reconstruction aims to restore the function of the ALL, thereby improving overall knee stability during dynamic activities. Both techniques have been associated with better alignment and reduced anterior tibial translation, which is crucial for knee function [33]. Complications, such as infection and graft failure, are significant considerations in ACL reconstruction. In some studies, reported graft failure rates can be as high as 10% to 13%, with factors such as graft choice, fixation methods, and surgical technique affecting these outcomes [34]. While LEAT and ALL reconstruction techniques may help reduce the risk of graft failure by providing additional stability, they also introduce potential complications, including longer surgical time and the risk of additional surgical sites [35]. Rehabilitation protocols vary depending on the surgical technique employed. LEAT generally requires a longer rehabilitation period due to the additional procedure involved. Conversely, ALL reconstruction may allow for a more accelerated rehabilitation timeline, often less invasive than LEAT. Nevertheless, both techniques require individualized rehabilitation plans tailored to the athlete’s needs and activity levels [36]. Recovery time can differ based on the complexity of the surgical procedure. Athletes undergoing ACL reconstruction with additional procedures like LEAT may experience longer recovery times due to the need for more comprehensive rehabilitation. In contrast, those who undergo isolated ALL reconstruction may return quicker to sport, although this also depends on adherence to rehabilitation protocols and overall health [37]. A comparative overview of ACL reconstruction techniques, including clinical outcomes, biomechanics, risks, and recovery, is summarized in Table 2.

**Table 2:** Comparison of ACL reconstruction techniques: clinical outcomes, biomechanics, risks, and recovery ACL, anterior cruciate ligament; ALL, anterolateral ligament; LEAT, lateral extra-articular tenodesis

Aspect	Traditional ACL reconstruction	LEAT	ALL reconstruction
Clinical outcomes [38]	High rates of return to sport, improved knee stability, but moderate risk of re-injury in pivoting sports.	Enhanced stability for patients with high rotational instability or pivot shift; reduces re-injury rates.	Better rotational stability in combination with ACL reconstruction is promising in reducing re-injury rates in pivot sports.
Biomechanical considerations [39]	Focuses primarily on anterior-posterior stability of the knee. It may not fully address rotational instability.	It provides additional rotational stability by reinforcing lateral structures and improving knee alignment.	Targets anterior-posterior and rotational stability, enhancing overall knee control and reducing pivot shift.
Complications and risks [40]	Risks include graft failure, infection, stiffness, and knee instability. Risk of damage to surrounding structures (e.g., patellar tendon).	Increased risk of over-constraining the lateral compartment; potential for joint stiffness and lateral knee pain.	Risk of complications similar to LEAT, including lateral knee pain, joint stiffness, and infection.
Rehabilitation and recovery [41]	Standard ACL rehab protocol focuses on restoring range of motion and strength and promoting a gradual return to activities.	Slightly prolonged recovery due to the additional lateral procedure; longer time to regain full rotational control.	Rehabilitation is similar to LEAT; however, return to sport may be faster than LEAT alone due to improved rotational control.
Return to sport rates [42]	High, though re-injury rates in pivot sports remain a concern.	Lower re-injury rates in pivot sports due to improved rotational stability.	Promising return-to-sport outcomes with lower re-injury rates, particularly in sports requiring pivot movements.

Considerations for young athletes

When addressing ACL injuries in young athletes, several critical factors must be considered, including the implications for growth plates, long-term outcomes, and the psychological and social impacts of the injury and recovery [43]. In young athletes, open growth plates present unique challenges for surgical intervention. Traditional ACL reconstruction methods often involve drilling tunnels through the growth plate, which can lead to complications such as stunted growth, leg length discrepancies, or angular deformities. To address these risks, newer surgical techniques that spare the growth plate have been developed [44]. For example, the all-epiphyseal technique places grafts entirely within the epiphysis, avoiding disruption of the growth plate. This approach has demonstrated promising results in maintaining normal growth patterns while effectively stabilizing the knee. Surgeons typically categorize young patients based on their skeletal maturity, selecting techniques according to the remaining growth potential. Pediatric surgical techniques are generally recommended for prepubertal children, while older adolescents may undergo transphyseal procedures as their growth plates approach closure [45]. Young athletes who undergo ACL reconstruction are at an increased risk of secondary injuries, including re-injury of the ACL itself and damage to associated structures like the meniscus. Research indicates that re-injury risk can be as high as 30% for young athletes returning to competitive sports [46]. Additionally, the long-term consequences of ACL injuries may include the early onset of osteoarthritis, especially if the initial injury is inadequately treated or if the surgical technique disrupts normal growth patterns. Therefore, carefully considering surgical techniques and rehabilitation protocols is crucial to minimizing these risks and promoting long-term joint health [7]. The psychological impact of injuries on young athletes can be significant. The pressure to return to sport, coupled with the fear of re-injury, can lead to anxiety and reduced confidence in their physical abilities. The social effects of being sidelined - such as the loss of camaraderie with teammates and decreased participation in social activities - can further contribute to feelings of isolation and depression [47]. Effective communication with athletes and their families about the recovery process, expectations, and the importance of rehabilitation is essential in mitigating these psychological impacts [47]. Additionally, involving sports psychologists in the recovery process can be beneficial in addressing the mental health aspects associated with ACL injuries and ensuring a comprehensive approach to recovery [47]. Considerations for ACL reconstruction in young athletes, including growth, long-term outcomes, and rehabilitation, are summarized in Table 3.

**Table 3:** Key considerations for ACL reconstruction in young athletes: growth, long-term outcomes, and rehabilitation ACL, anterior cruciate ligament; ALL, anterolateral ligament; LEAT, lateral extra-articular tenodesis

Consideration	Traditional ACL reconstruction	LEAT	ALL reconstruction
Growth plate concerns [48]	Risk of growth plate damage, especially in skeletally immature athletes. Requires specialized techniques to avoid growth disturbance.	Generally, it is not recommended in skeletally immature athletes due to the risk of growth plate injury.	Risk of damaging growth plates, particularly with lateral structure involvement.
Long-term outcomes [49]	Good long-term outcomes but higher re-injury rates in sports involving pivoting. Potential need for revision surgery later.	Better long-term rotational stability, reducing the likelihood of re-injury, particularly in pivot-heavy sports.	Promising long-term outcomes with improved rotational control, reducing the likelihood of re-injury.
Psychological impact [50]	A long rehabilitation process may impact the athlete’s motivation and mental health. Return-to-sport anxiety is common.	Longer recovery time may increase psychological stress and slower return to sports.	A faster return to sport can positively impact mental well-being and confidence in performance.
Risk of secondary injuries [51]	Moderate risk of secondary injuries, especially if rotational instability persists after surgery.	Lower risk of secondary injuries due to improved rotational stability and decreased pivot shift.	Reduced risk of secondary injuries, especially in pivot-heavy sports, due to better control of knee movements.
Rehabilitation protocol [52]	Standard ACL protocol focused on strength, balance, and gradual return to activity. Takes around 9-12 months for full recovery.	Longer rehab due to additional procedures; full recovery can take up to 12-18 months.	Rehab is similar to LEAT, but return-to-sport may be quicker due to better rotational control.
Return to competitive sports [53]	High, though some athletes may not regain pre-injury performance levels. The risk of re-injury may deter some from returning to high-level sports.	Good return-to-sport outcomes, particularly in high-demand sports requiring rotational stability.	High return-to-sport rates with improved knee stability and reduced re-injury risk.

Future directions and research

The ACL reconstruction field is advancing rapidly, with new techniques and ongoing research aimed at improving patient outcomes, particularly for young athletes. This overview highlights recent innovations in surgical techniques and identifies areas requiring further investigation [54]. Emerging techniques include all-inside ACL reconstruction, which employs retrograde drilling to create bony sockets for graft passage. This approach aims to reduce complications, minimize pain, and enhance recovery while preserving bone stock. Studies indicate promising clinical outcomes and graft survival rates with this method, making it a viable option for various patient groups, including adolescents [35]. Dynamic intraligamentary stabilization (DIS) techniques, such as the Ligamys™ system (Ligamys, Bettlach, Switzerland) and bridge-enhanced ACL repair (BEAR), are also gaining attention. These methods focus on preserving the native ACL structure while enhancing stability through innovative devices and bioactive scaffolds, reflecting a growing interest in primary ACL repair strategies. Double-bundle ACL reconstruction aims to restore the anatomical configuration of the ACL by recreating both the AM and PL bundles. This technique has improved stability and function compared to single-bundle methods, particularly during dynamic activities [55]. The independent drilling technique allows for more anatomically accurate tunnel placement during reconstruction, associated with better knee stability and a reduced risk of postoperative osteoarthritis compared to traditional transtibial drilling techniques [56]. Despite the promise of these new techniques, there is a critical need for long-term studies to assess their effectiveness and durability. Most current research focuses on short- to medium-term outcomes, leaving a gap in understanding the long-term implications of these techniques on knee stability and function. Additional research is needed to compare the effectiveness of emerging techniques with established methods [57]. Understanding each approach's relative benefits and risks will guide clinical decision-making and optimize patient outcomes. This includes comparing graft types, surgical techniques, and rehabilitation protocols to identify best practices for specific patient populations, especially young athletes at higher risk of reinjury [58]. It is crucial for pediatric and adolescent patients to study how various surgical techniques impact growth and development. Techniques, such as all-epiphyseal ACL reconstruction, aim to minimize the effects on growth plates, but comprehensive studies are necessary to ensure their safety and efficacy in growing patients [59]. Future directions and research in ACL reconstruction, addressing current limitations and advancing techniques, are outlined in Table 4.

**Table 4:** Future directions and research in ACL reconstruction: addressing current limitations and advancing techniques ACL, anterior cruciate ligament; ALL, anterolateral ligament; LEAT, lateral extra-articular tenodesis

Research area	Current limitations	Future directions	Potential impact
Long-term outcomes of LEAT and ALL [60]	Limited long-term data on the effectiveness and durability of LEAT and ALL techniques.	Conducting large-scale, long-term studies to assess the durability and effectiveness of LEAT and ALL techniques over several decades.	Better understanding of the long-term success rates, reducing re-injury rates, and improving treatment protocols.
Growth plate-sparing techniques [61]	Current techniques pose risks to growth plates in skeletally immature athletes.	Development of growth plate-sparing surgical techniques to prevent growth disturbance.	Improved safety of ACL reconstructions in younger populations with minimal impact on growth and development.
Biomechanical studies on rotational stability [62]	Limited biomechanical evidence on the superiority of LEAT and ALL in restoring rotational stability.	More advanced biomechanical studies using real-time imaging and computational models to evaluate rotational stability after reconstruction.	Enhanced precision in surgical techniques and improved rotational stability and knee function outcomes.
Rehabilitation optimization [63]	Standard rehabilitation protocols may not address individual needs, especially for high-performance athletes.	Tailoring rehabilitation protocols based on patient-specific factors such as age, activity level, and psychological readiness.	Faster recovery, improved return-to-sport outcomes, and lower re-injury rates.
Psychological impact of ACL injuries [64]	There is limited focus on mental and emotional recovery post-ACL reconstruction, especially for young athletes.	Research on integrating psychological support and mental health interventions into ACL rehabilitation programs.	Improved mental well-being, motivation, and overall recovery experience for young athletes.
Innovative surgical techniques [65]	Current surgical approaches may have limitations in addressing complex ACL injuries.	Exploration of new surgical techniques, such as minimally invasive or robotic-assisted ACL reconstruction.	Potential to reduce recovery time, lower complication rates, and improve precision in ACL reconstructions.

## Conclusions

In conclusion, managing ACL injuries in young athletes presents significant challenges due to the high physical demands and long-term implications associated with these injuries. This review has highlighted the advancements in surgical techniques, specifically LEAT and ALL reconstruction, as potential solutions to enhance outcomes in ACL reconstruction. By providing a comparative analysis of these techniques, the review underscores the importance of tailored approaches to treatment that address not only immediate stability but also long-term joint health and athletic performance. Both LEAT and ALL reconstruction offer promising benefits, but their effectiveness can vary based on individual patient factors and the specific demands of their sport. Further research and long-term studies are needed to refine these techniques and better understand their impact on recovery and prevention of future complications. Ultimately, the goal is to provide young athletes with the best possible outcomes, ensuring they can return to their sport with confidence and optimal knee function.

## References

[1] Joseph AM, Collins CL, Henke NM, Yard EE, Fields SK, Comstock RD (2013). A multisport epidemiologic comparison of anterior cruciate ligament injuries in high school athletics. J Athl Train.

[2] Bram JT, Magee LC, Mehta NN, Patel NM, Ganley TJ (2021). Anterior cruciate ligament injury incidence in adolescent athletes: a systematic review and meta-analysis. Am J Sports Med.

[3] Amoako AO, Pujalte GG (2014). Osteoarthritis in young, active, and athletic individuals. Clin Med Insights Arthritis Musculoskelet Disord.

[4] Joreitz R, Lynch A, Rabuck S, Lynch B, Davin S, Irrgang J (2016). Patient-specific and surgery-specific factors that affect return to sport after ACL reconstruction. Int J Sports Phys Ther.

[5] Abusleme S, Strömbäck L, Caracciolo G, Zamorano H, Cheyre J, Vergara F, Yañez R (2021). Lateral extra-articular tenodesis: a technique with an iliotibial band strand without implants. Arthrosc Tech.

[6] Domnick C, Raschke MJ, Herbort M (2016). Biomechanics of the anterior cruciate ligament: physiology, rupture and reconstruction techniques. World J Orthop.

[7] Evans J, Mabrouk A, Nielson J l (2024). Anterior cruciate ligament knee injury. StatPearls.

[8] Yaras RJ, O’Neill N, Mabrouk A, Yaish AM (2024). Lateral collateral ligament knee injury. StatPearls.

[9] Ahn JH, Patel NA, Lin CC, Lee TQ (2019). The anterolateral ligament of the knee joint: a review of the anatomy, biomechanics, and anterolateral ligament surgery. Knee Surg Relat Res.

[10] Willinger L, Athwal KK, Holthof S, Imhoff AB, Williams A, Amis AA (2023). Role of the anterior cruciate ligament, anterolateral complex, and lateral meniscus posterior root in anterolateral rotatory knee instability: a biomechanical study. Am J Sports Med.

[11] Guarino A, Farinelli L, Iacono V (2022). Lateral extra-articular tenodesis and anterior cruciate ligament reconstruction in young patients: clinical results and return to sport. Orthop Rev (Pavia).

[12] Murawski CD, Wolf MR, Araki D, Muller B, Tashman S, Fu FH (2013). Anatomic anterior cruciate ligament reconstruction: current concepts and future perspective. Cartilage.

[13] Runer A, Keeling L, Wagala N, Nugraha H, Özbek EA, Hughes JD, Musahl V (2023). Current trends in graft choice for primary anterior cruciate ligament reconstruction - part II: In-vivo kinematics, patient reported outcomes, re-rupture rates, strength recovery, return to sports and complications. J Exp Orthop.

[14] Devitt BM, Bell SW, Ardern CL, Hartwig T, Porter TJ, Feller JA, Webster KE (2017). The role of lateral extra-articular tenodesis in primary anterior cruciate ligament reconstruction: a systematic review with meta-analysis and best-evidence synthesis. Orthop J Sports Med.

[15] Sim K, Rahardja R, Zhu M, Young SW (2022). Optimal graft choice in athletic patients with anterior cruciate ligament injuries: review and clinical insights. Open Access J Sports Med.

[16] Kawanishi Y, Kobayashi M, Yasuma S (2021). Anterolateral ligament reconstruction in addition to primary double-bundle anterior cruciate ligament reconstruction for grade 3 pivot shift improves residual knee instability during surgery. J Exp Orthop.

[17] Rodriguez K, Soni M, Joshi PK (2021). Anterior cruciate ligament injury: conservative versus surgical treatment. Cureus.

[18] Jenkins SM, Guzman A, Gardner BB, Bryant SA, Del Sol SR, McGahan P, Chen J (2022). Rehabilitation after anterior cruciate ligament injury: review of current literature and recommendations. Curr Rev Musculoskelet Med.

[19] Meena A, Das S, Runer A (2024). Revision ACL reconstruction in female athletes: current concepts. J ISAKOS.

[20] Smeets A, Ghafelzadeh Ahwaz F, Bogaerts S, Berger P, Peers K (2024). Comparison of immediate versus optional delayed surgical repair for treatment of acute anterior cruciate ligament injury through a parallel, multicentric, pragmatic randomized controlled trial - IODA trial. BMC Sports Sci Med Rehabil.

[21] Piedade SR, Leite Arruda BP, de Vasconcelos RA, Parker DA, Maffulli N (2023). Rehabilitation following surgical reconstruction for anterior cruciate ligament insufficiency: what has changed since the 1960s?—state of the art. J ISAKOS.

[22] Wilk KE, Macrina LC, Cain EL, Dugas JR, Andrews JR (2012). Recent advances in the rehabilitation of anterior cruciate ligament injuries. J Orthop Sports Phys Ther.

[23] Mahapatra P, Horriat S, Anand BS (2018). Anterior cruciate ligament repair - past, present and future. J Exp Orthop.

[24] Nukuto K, Hoshino Y, Kataoka K, Kuroda R (2023). Current development in surgical techniques, graft selection and additional procedures for anterior cruciate ligament injury: a path towards anatomic restoration and improved clinical outcomes—a narrative review. Ann Jt.

[25] Cohen D, Slawaska-Eng D, Almasri M, Sheean A, de Sa D (2021). Quadricep ACL reconstruction techniques and outcomes: an updated scoping review of the quadricep tendon. Curr Rev Musculoskelet Med.

[26] Bernholt DL, Kennedy MI, Crawford MD, DePhillipo NN, LaPrade RF (2019). Combined anterior cruciate ligament reconstruction and lateral extra-articular tenodesis. Arthrosc Tech.

[27] Santos DA, Rocha de Faria JL, Carminatti T (2023). Combined all-inside anterior cruciate ligament reconstruction with semitendinosus plus anterolateral ligament reconstruction with intact gracilis tibial insertion and transtibial passage. Arthrosc Tech.

[28] Lai S, Zhang Z, Li J, Fu WL (2023). Comparison of anterior cruciate ligament reconstruction with versus without anterolateral augmentation: a systematic review and meta-analysis of randomized controlled trials. Orthop J Sports Med.

[29] Mardani-Kivi M, Leili EK, Shirangi A, Azari Z (2021). Return to sports activity in the revision of anterior cruciate ligament reconstruction: a 2-6 Year follow-up study. J Clin Orthop Trauma.

[30] Buerba RA, Zaffagnini S, Kuroda R, Musahl V (2021). ACL reconstruction in the professional or elite athlete: state of the art. J ISAKOS.

[31] Buckthorpe M, La Rosa G, Villa FD (2019). Restoring knee extensor strength after anterior cruciate ligament reconstruction: a clinical commentary. Int J Sports Phys Ther.

[32] Park JG, Han SB, Lee CS, Jeon OH, Jang KM (2022). Anatomy, biomechanics, and reconstruction of the anterolateral ligament of the knee joint. Medicina (Kaunas).

[33] Tulloch S, Getgood A (2019). Consideration of lateral augmentation in anatomic anterior cruciate ligament reconstruction. Ann Joint.

[34] Samitier G, Marcano AI, Alentorn-Geli E, Cugat R, Farmer KW, Moser MW (2015). Failure of anterior cruciate ligament reconstruction. Arch Bone Jt Surg.

[35] Bosco F, Giustra F, Ghirri A, Cacciola G, Massè A, Capella M (2023). All-inside anterior cruciate ligament reconstruction technique: tips and tricks. J Clin Med.

[36] Wu J, Kator JL, Zarro M, Leong NL (2022). Rehabilitation principles to consider for anterior cruciate ligament repair. Sports Health.

[37] Brinlee AW, Dickenson SB, Hunter-Giordano A, Snyder-Mackler L (2022). ACL reconstruction rehabilitation: clinical data, biologic healing, and criterion-based milestones to inform a return-to-sport guideline. Sports Health.

[38] Zanna L, Niccolò G, Matteo I, Malone J, Roberto C, Fabrizio M (2023). Clinical outcomes and return to sport after single-stage revision anterior cruciate ligament reconstruction by bone-patellar tendon autograft combined with lateral extra-articular tenodesis. Eur J Orthop Surg Traumatol.

[39] Zlotnicki JP, Naendrup JH, Ferrer GA, Debski RE (2016). Basic biomechanic principles of knee instability. Curr Rev Musculoskelet Med.

[40] Marshall DC, Silva FD, Goldenberg BT, Quintero D, Baraga MG, Jose J (2022). Imaging findings of complications after lateral extra-articular tenodesis of the knee: a current concepts review. Orthop J Sports Med.

[41] Bousquet BA, O'Brien L, Singleton S, Beggs M (2018). Post-operative criterion based rehabilitation of ACL repairs: a clinical commentary. Int J Sports Phys Ther.

[42] Bitar AC, Scalize AR, Abreu G, D'Elia C, Ribas LH, Castropil W (2021). Return to sport and re-injury rate after double-bundle anterior cruciate ligament reconstruction with at least five years of follow-up. Arch Bone Jt Surg.

[43] Vutescu ES, Orman S, Garcia-Lopez E, Lau J, Gage A, Cruz AI Jr (2021). Psychological and social components of recovery following anterior cruciate ligament reconstruction in young athletes: a narrative review. Int J Environ Res Public Health.

[44] Kercher J, Xerogeanes J, Tannenbaum A, Al-Hakim R, Black JC, Zhao J (2009). Anterior cruciate ligament reconstruction in the skeletally immature: an anatomical study utilizing 3-dimensional magnetic resonance imaging reconstructions. J Pediatr Orthop.

[45] Pennock AT, Chambers HG, Turk RD, Parvanta KM, Dennis MM, Edmonds EW (2018). Use of a modified all-epiphyseal technique for anterior cruciate ligament reconstruction in the skeletally immature patient. Orthop J Sports Med.

[46] Wiggins AJ, Grandhi RK, Schneider DK, Stanfield D, Webster KE, Myer GD (2016). Risk of secondary injury in younger athletes after anterior cruciate ligament reconstruction: a systematic review and meta-analysis. Am J Sports Med.

[47] Juggath C, Naidoo R (2024). The influence of psychological readiness of athletes when returning to sport after injury. S Afr J Sports Med.

[48] Caine D, Purcell L, Maffulli N (2014). The child and adolescent athlete: a review of three potentially serious injuries. BMC Sports Sci Med Rehabil.

[49] Dahduli OS, AlHossan AM, Al Rushud MA (2023). Early surgical reconstruction versus rehabilitation for patients with anterior cruciate ligament rupture: a systematic review and meta-analysis. Cureus.

[50] Monsma E, Mensch J, Farroll J (2009). Keeping your head in the game: sport-specific imagery and anxiety among injured athletes. J Athl Train.

[51] Diermeier T, Rothrauff BB, Engebretsen L (2020). Treatment after anterior cruciate ligament injury: Panther Symposium ACL Treatment Consensus Group. Knee Surg Sports Traumatol Arthrosc.

[52] Cunha J, Solomon DJ (2022). ACL prehabilitation improves postoperative strength and motion and return to sport in athletes. Arthrosc Sports Med Rehabil.

[53] Drole K, Paravlic AH (2022). Interventions for increasing return to sport rates after an anterior cruciate ligament reconstruction surgery: a systematic review. Front Psychol.

[54] Tang C, Kwaees TA, Accadbled F, Turati M, Green DW, Nicolaou N (2023). Surgical techniques in the management of pediatric anterior cruciate ligament tears: current concepts. J Child Orthop.

[55] Malahias MA, Chytas D, Nakamura K, Raoulis V, Yokota M, Nikolaou VS (2018). A narrative review of four different new techniques in primary anterior cruciate ligament repair: “back to the future” or another trend?. Sports Med Open.

[56] Cuzzolin M, Previtali D, Delcogliano M, Filardo G, Candrian C, Grassi A (2021). Independent versus transtibial drilling in anterior cruciate ligament reconstruction: a meta-analysis with meta-regression. Orthop J Sports Med.

[57] Abernethy A, Adams L, Barrett M (2022). The promise of digital health: then, now, and the future. NAM Perspect.

[58] Leung A, DeSandis B, O'Brien L, Hammoud S, Zarzycki R (2023). Postoperative considerations based on graft type after anterior cruciate ligament reconstruction a narrative review. Ann Jt.

[59] Pennock A, Murphy MM, Wu M (2016). Anterior cruciate ligament reconstruction in skeletally immature patients. Curr Rev Musculoskelet Med.

[60] Courcoulas AP, Yanovski SZ, Bonds D, Eggerman TL, Horlick M, Staten MA, Arterburn DE (2014). Long-term outcomes of bariatric surgery: a National Institutes of Health symposium. JAMA Surg.

[61] Willson RG, Kostyun RO, Milewski MD, Nissen CW (2018). Anterior cruciate ligament reconstruction in skeletally immature patients: early results using a hybrid physeal-sparing technique. Orthop J Sports Med.

[62] Oliveira D Elia C, Bitar AC, Orselli MI, Castropil W, Duarte M, Camanho G (2022). Rotational stability of the knee in a comparative study of anterior cruciate ligament reconstruction using the double-bundle and single-bundle techniques. Arch Bone Jt Surg.

[63] Badawy CR, Jan K, Beck EC, Fleet N, Taylor J, Ford K, Waterman BR (2022). Contemporary principles for postoperative rehabilitation and return to sport for athletes undergoing anterior cruciate ligament reconstruction. Arthrosc Sports Med Rehabil.

[64] Nyland J, Pyle B (2022). Self-identity and adolescent return to sports post-acl injury and rehabilitation: will anyone listen?. Arthrosc Sports Med Rehabil.

[65] Raines BT, Naclerio E, Sherman SL (2017). Management of anterior cruciate ligament injury: what’s in and what’s out?. Indian J Orthop.

